# Purification and characterization of a novel medium-chain ribitol dehydrogenase from a lichen-associated bacterium *Sphingomonas* sp.

**DOI:** 10.1371/journal.pone.0235718

**Published:** 2020-07-08

**Authors:** Kiet N. Tran, Nhung Pham, Sei-Heon Jang, ChangWoo Lee

**Affiliations:** Department of Biomedical Science and Center for Bio-Nanomaterials, Daegu University, Gyeongsan, South Korea; Luleå University of Technology, SWEDEN

## Abstract

Sugar alcohols (polyols) are abundant carbohydrates in lichen-forming algae and transported to other lichen symbionts, fungi, and bacteria. Particularly, ribitol is an abundant polyol in the lichen *Cetraria* sp. Polyols have important physiological roles in lichen symbiosis, but polyol utilization in lichen-associated bacteria has been largely unreported. Herein, we purified and characterized a novel ribitol dehydrogenase (RDH) from a *Cetraria* sp.-associated bacterium *Sphingomonas* sp. PAMC 26621 grown on a minimal medium containing D-ribitol (the RDH hereafter referred to as SpRDH). SpRDH is present as a trimer in its native form, and the molecular weight of SpRDH was estimated to be 39 kDa by SDS-PAGE and 117 kDa by gel filtration chromatography. SpRDH converted D-ribitol to D-ribulose using NAD^+^ as a cofactor. As far as we know, SpRDH is the first RDH belonging to the medium-chain dehydrogenase/reductase family. Multiple sequence alignments indicated that the catalytic amino acid residues of SpRDH consist of Cys37, His65, Glu66, and Glu157, whereas those of short-chain RDHs consist of Ser, Tyr, and Lys. Furthermore, unlike other short-chain RDHs, SpRDH did not require divalent metal ions for its catalytic activity. Despite SpRDH originating from a psychrophilic Arctic bacterium, *Sphingomonas* sp., it had maximum activity at 60°C and exhibited high thermal stability within the 4–50°C range. Further studies on the structure/function relationship and catalytic mechanism of SpRDH will expand our understanding of its role in lichen symbiosis.

## Introduction

Lichens have traditionally been considered a symbiotic association between fungi (mycobionts) and either algae or cyanobacteria (photobionts). However, recent studies demonstrated that non-photosynthetic bacteria are also an integral part of lichens [1–4]. In lichens, photobionts synthesize and transport carbohydrates to mycobionts and bacteria; specifically, cyanobacteria release glucose while green algae liberate sugar alcohols (polyols) such as D-ribitol, Meso-erythritol, and D-sorbitol [5, 6]. In contrast, fungi and bacteria supply nutrients (nitrogen, phosphorus, iron, and sulfur) and vitamins to photobionts [7, 8].

Polyols have a role in carbohydrate storage, and they protect organisms from osmotic, salt, and oxidative stresses [9, 10]. Polyols also protect lichens in cold habitats, acting as a cryoprotectant [11]. D-ribitol and D-mannitol are the most abundant polyols in lichens [12]. D-ribitol is exported from algal photobionts to mycobionts [5], whereas D-mannitol is produced and metabolized by lichen fungi [13]. The lichen-associated bacterium *Sphingomonas* sp. TZW 2008 can grow on D-ribitol and D-mannitol [14]. Unlike sugars that directly enter the metabolic pathways, polyols require a combination of dehydrogenase and kinase before entering pathways in which polyol dehydrogenases convert between rare sugar and its sugar alcohol form [15]. Among the polyol dehydrogenases, mannitol dehydrogenase, sorbitol dehydrogenase (SDH), xylitol dehydrogenase (XDH), and ribitol dehydrogenase (RDH) have been studied the most [15]. SDH belongs to either the short-chain dehydrogenase/reductase (SDR) family (25–30 kDa) or the medium-chain dehydrogenase/reductase (MDR) family (30–40 kDa) [16, 17], and D-sorbitol is converted to D-fructose by SDH, whereas D-ribitol is converted to D-ribulose by RDH. All reported RDHs belong to the SDR family [18–20].

In the present study, we sought to elucidate polyol utilization in *Sphingomonas* sp. PAMC 26621, a bacterium isolated from the Arctic lichen *Cetraria* sp. [21]. Based on the observation that *Sphingomonas* sp. PAMC 26621 grew only on a minimal medium containing D-ribitol as a sole carbon source among the polyols investigated (Meso-erythritol, D-mannitol, D-ribitol, D-sorbitol, or D-xylitol), we purified and characterized a novel RDH (SpRDH) from *Sphingomonas* sp. PAMC 26621. To the best of our knowledge, SpRDH is the first medium-chain RDH in which the catalytic amino acid residues are different from those of short-chain RDHs, based on multiple sequence alignment results. Furthermore, SpRDH does not require divalent metal ions for its catalytic activity.

## Materials and methods

### Materials

*Sphingomonas* sp. PAMC 26621 was kindly provided by the Polar and Alpine Microbial Collection of the Korea Polar Research Institute (Incheon, South Korea) [22]. HiPrep DEAE FF 16/10, HiPrep SP FF 16/10, Mono Q HR 5/5, Superdex 200 10/300 GL, and HiTrap desalting 26/10 columns were purchased from GE Healthcare (Piscataway, NJ, USA). Amicon Ultra centrifugal filters (10 kDa) were obtained from Merck Millipore (Carrigtwohill, Ireland). All other reagents were acquired from Sigma (St. Louis, MO, USA) unless otherwise stated.

### Growth of *Sphingomonas* sp. PAMC 26621 on different carbon sources

An individual colony of *Sphingomonas* sp. PAMC 26621 grown on a Reasoner’s 2A plate was inoculated into Reasoner’s 2A broth and cultivated for two days at 15°C (OD_600_ = 0.8–1.0). Next, 1 mL of the culture medium was transferred into 100 mL of minimal medium (10.5 g L^-1^ K_2_HPO_4_, 5 g L^-1^ KH_2_PO_4_, 1 g L^-1^ (NH_4_)_2_SO_4_, 0.5 g L^-1^ sodium citrate, 2 mM MgSO_4_, 15 μM vitamin B_1_, and 1% (w/v) carbon source) containing D-glucose, D-ribitol, D-xylitol, D-mannitol, Meso-erythritol, or D-sorbitol as the sole carbon source. The cultures were grown at 15°C with shaking at 225 rpm, and every 24 h, the cell density of the cultures was determined based on the absorbance at 600 nm by using a Shimadzu UV-1800 spectrophotometer.

### Purification of SpRDH

*Sphingomonas* sp. PAMC 26621 was cultured in minimal medium containing 1% (w/v) D-ribitol at 15°C, after which the cells were harvested by centrifugation at 10,000 × *g* for 20 min and suspended in buffer A (50 mM Tris·HCl, pH 8.0, 50 mM KCl). Proteins were extracted by using sonication, and the cell lysate was collected by centrifugation at 20,000 × *g* for 30 min. The cell lysate was then precipitated by ammonium sulfate, after which the fraction containing 30–60% saturated ammonium sulfate was collected by centrifugation at 20,000 × *g* for 30 min. The pellet was resuspended and desalted in buffer A by using a HiTrap desalting 26/10 column. The eluates were applied to a HiPrep DEAE FF 16/10 column equilibrated with buffer A and using a flow rate of 2 mL/min. Proteins were subsequently eluted using a KCl gradient (50–200 mM KCl with two column volumes [CV], 200–500 mM with 15 CV, and 500–1000 mM KCl with 3 CV). Active fractions were pooled and desalted in buffer B (50 mM sodium acetate, pH 5.0, 50 mM NaCl) in a desalting column, then loaded into a HiPrep SP FF 16/10 column equilibrated with buffer B. Proteins were eluted by a linear gradient of 50–1000 mM NaCl in buffer B at a flow rate of 2 mL/min. Active fractions were pooled and immediately desalted in buffer C (20 mM L-Histidine, pH 6.0, 50 mM KCl) using a desalting column. This sample was then applied to a Mono Q HR 5/5 column (1 mL) equilibrated with buffer C, after which the proteins were eluted in a KCl gradient in buffer C (50–200 mM KCl, 5 CV; 200–400 mM KCl, 20 CV; 400–1000 mM KCl, 5 CV) at a flow rate of 0.5 mL/min. Active fractions were pooled and desalted in buffer A using a 5 mL-Amicon Ultra centrifugal filter (10 kDa) followed by purification using a Mono Q column with buffer A, after which the proteins were eluted out under the same conditions mentioned above for the initial Mono Q column purification, except that buffer A was used as the elution buffer.

All steps of the purification process were conducted at 4°C and products were analyzed by SDS-PAGE, with the chromatographic steps performed on an AKTA Explorer system (GE Healthcare).

### Macromolecular complex analysis

The molecular weight of native SpRDH was determined by performing gel filtration chromatography and native-PAGE. Standard proteins (ovalbumin [44 kDa], BSA [66 kDa], glutathione reductase [100 kDa], and catalase [232 kDa]) were dissolved in buffer D (50 mM sodium phosphate, pH 7.2, 150 mM NaCl) and then applied to a Superdex 200 10/300 GL column equilibrated with buffer D. The proteins were eluted by buffer D at a flow rate of 0.2 mL/min after which purified SpRDH (1 mL in buffer D) was applied to the column and eluted under the same conditions as described above. The native molecular weight of the enzyme was calculated from a standard graph plot based on the molecular weight (kDa) of the standard proteins against their elution volume (mL). Native-PAGE was conducted as previously described [23] using glutathione reductase and BSA as standard proteins.

### Enzyme assay

RDH activity was determined by measuring the absorbance of NADH at 340 nm on a Shimadzu UV-1800 spectrophotometer at 25°C. The reaction mixture (500 μL) contained 1 mM NAD^+^, 40 mM D-ribitol, and an appropriate amount of SpRDH in 100 mM potassium phosphate at pH 8.0. Measurements were performed during the first 2 min of the reaction at 25°C. One unit of SpRDH was defined as the amount of enzyme required to produce 1 μmol NADH per minute under the given assay conditions.

### Effects of pH and temperature on SpRDH activity

The optimum pH of SpRDH was determined by conducting an enzyme assay in a buffer system of 100 mM potassium phosphate (pH 6.0−8.0), and 50 mM Tris·HCl (pH 8.0–11.0). The apparent optimal temperature was determined by measuring the enzyme activity at 10−80°C, as described above in the Enzyme assay.

The thermal stability of SpRDH was determined by measuring the residual activity level, as described in the Enzyme assay. The SpRDH enzyme was incubated at various temperatures (4°C, 25°C, 40°C, 50°C, and 60°C). The temperature-treated enzyme samples were aliquoted every 30 min for up to 4 h to determine the activity level at 60°C. The activity of SpRDH at 60°C, before enzyme incubation, was considered to be 100%.

### Metal ions analysis

The concentration of metal ions (Zn^2+^, Mg^2+^, Mn^2+^, Cu^2+^, Fe^2+^, Ni^2+^, and Co^2+^) was measured at the Korea Basic Science Institute (Seoul, South Korea) by performing Inductively Coupled Plasma-Mass Spectrometry (ICP-MS) on a 7700x ICP-MS system (Agilent Technologies, Tokyo, Japan) equipped with a PFA micro-concentric nebulizer and a double pass spray chamber. The SpRDH (0.5 mg/mL) was prepared in Tris buffer (20 mM Tris·HCl, pH 8.0, 20 mM KCl) before measurement. Besides, the effect of EDTA on SpRDH activity was measured in phosphate buffer at pH 8.0.

### Kinetic assay

Kinetic parameters of SpRDH were determined by measuring the initial velocities of SpRDH at several fixed concentrations (10–160 mM) of D-ribitol when NAD^+^ concentration was varied within the range of 0.05–1 mM at 25°C, and vice versa. The data were analyzed using the SigmaPlot Enzyme Kinetic Module on SigmaPlot 14.0 (Systat Software Inc., San Jose, CA, USA). All experiments were conducted three times.

### Identification of SpRDH-catalyzed reaction by HPLC

The product of the SpRDH oxidation reaction was identified by using HPLC. Briefly, 1 mL of reaction mixture containing 5 mM NAD^+^, 100 mM D-ribitol, and 0.06 μM SpRDH in 100 mM potassium phosphate at pH 8.0 was incubated at 40°C for 2 h. Subsequently, the reaction mixture was loaded onto a Sugar-Pak I column (Ø 6.5 × 300 mm) using a Waters Alliance HPLC system. The substances were eluted by using a mobile phase of 10 mM Ca-EDTA at 90°C at a flow rate of 0.5 mL/min. Peaks were detected by a refractive index detector, with 10 mg/ml of D-ribulose, D-ribose, D-ribitol, and NAD^+^ used to generate authentic graphs.

### Proteomic analysis

Purified SpRDH enzyme was identified by matrix-assisted laser desorption/ionization time-of-flight mass spectroscopy (MALDI-TOF/TOF) undertaken at Genomine (Pohang, South Korea). The trypsin-digested protein samples of SpRDH were analyzed using an Autoflex Speed MALDI-TOF system (Bruker) with LIFTTM ion optics. Mass spectrum data were acquired using the instrument’s default calibration without applying internal or external calibration (S1A Fig). Peaks in the spectrum were selected for inclusion in a Mascot search by using Flexanalysis Biotool Mascot software. The MS/MS ion searches against those in the NCBI protein database were performed by using the Mascot program (S1B Fig). Similar searches were performed by applying the NCBI BLAST tool. Amino acid sequence alignments of SpRDH and other RDHs were performed by using Clustal Omega [24].

## Results

### Effects of various carbon sources on bacterial growth

**Fig 1:**
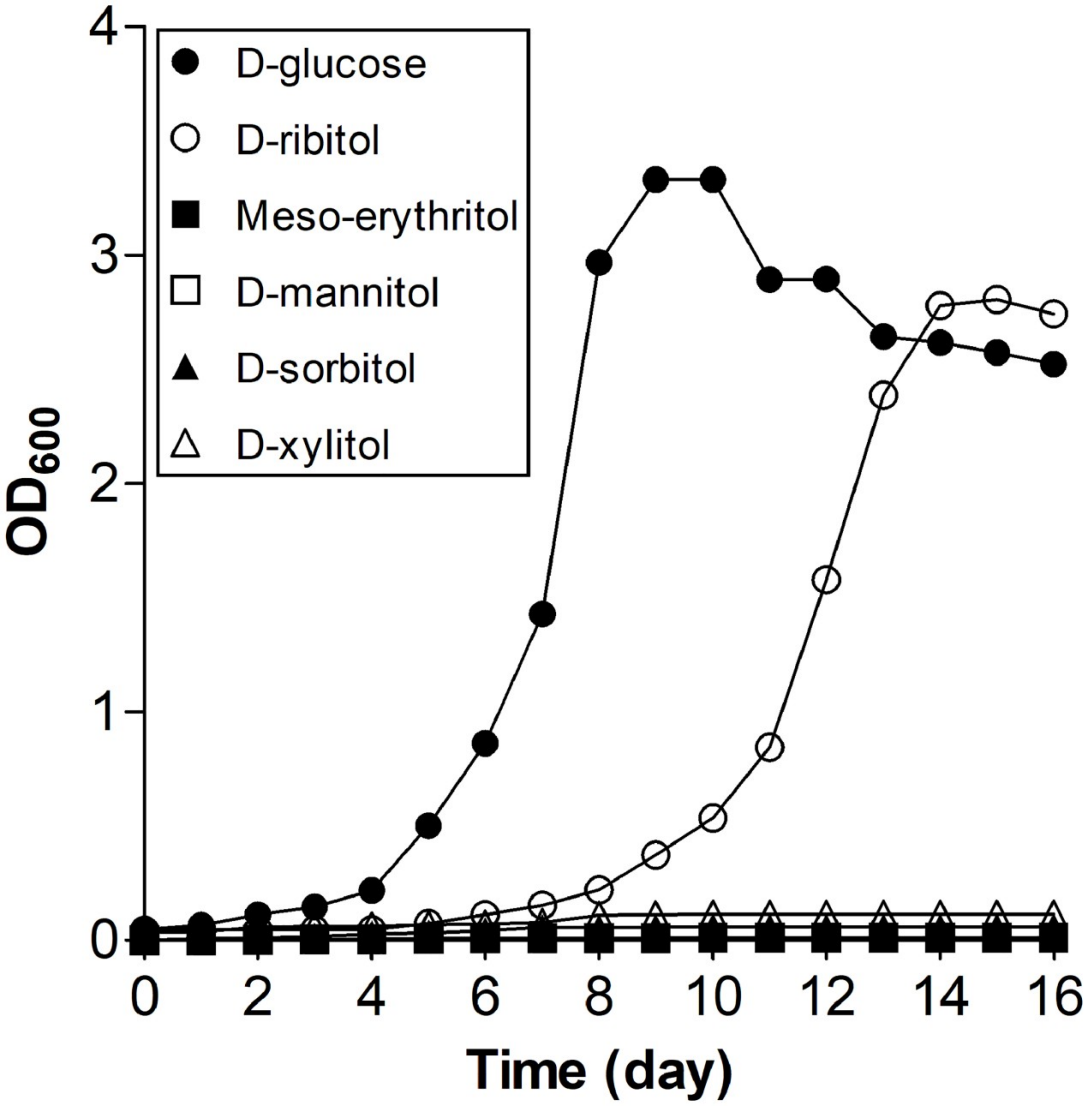
Effect of different carbon sources on the growth of *Sphingomonas* sp. PAMC 26621. The bacterium was cultured in minimal medium containing 1% (w/v) of different carbon sources (D-glucose, D-ribitol, D- xylitol, D-mannitol, D-sorbitol, or Meso-erythritol) at 15°C with shaking at 225 rpm. Cell densities were measured at OD_600_ at the indicated times.

*Sphingomonas* sp. PAMC 26621 was inoculated on a minimal medium containing either 1% (w/v) glucose or a polyol (D-ribitol, Meso-erythritol, D-xylitol, D-sorbitol, or D-mannitol) at 15°C and its growth measured for 16 days. The growth on D-glucose was the fastest (Fig 1). *Sphingomonas* sp. PAMC 26621 thrived only on D-ribitol among the examined polyols with a four-day longer lag phase compared with that on D-glucose but reached a similar plateau to that observed with D-glucose (Fig 1). Although D-mannitol and D-ribitol are, in general, the most abundant polyols in lichens [12, 25], the bacterium could not utilize D-mannitol for its growth (Fig 1). These data indicated that D-ribitol is the preferred polyol of *Sphingomonas* sp. PAMC 26621.

### Purification of SpRDH

**Fig 2:**
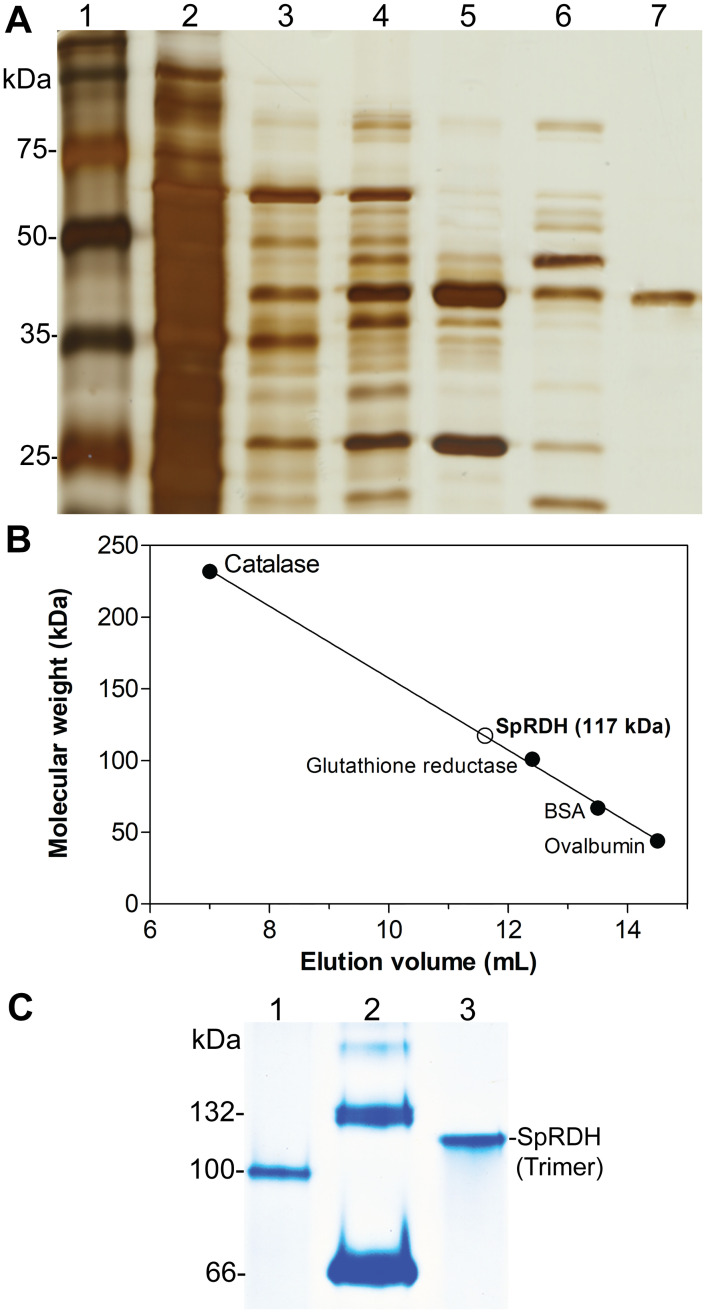
Purification and molecular weight determination of SpRDH. (A) Silver-stained SDS gel after protein purification. Lane 1, marker; Lane 2, ammonium sulfate precipitation (30–60%); Lane 3, DEAE elution fraction; Lane 4, SP elution fraction; Lanes 5 and 6, Mono Q elution fractions (pH 6.0); Lane 7, Mono Q elution fraction (pH 8.0). (B) Determination of the molecular weight of native SpRDH by performing Superdex 200 gel filtration chromatography. Catalase (232 kDa), glutathione reductase (100 kDa), BSA (66 kDa), and ovalbumin (44 kDa). (C) Native-PAGE analysis of SpRDH. Lane 1, glutathione reductase; Lane 2, BSA; Lane 3, SpRDH.

RDH activity was detected in the cell extract of *Sphingomonas* sp. PAMC 26621 grown on a minimal medium containing D-ribitol. However, a BLAST search using known RDH sequences as queries against the genome of *Sphingomonas* sp. PAMC 26621 (NCBI Accession No.: AIDW00000000.1) resulted in no annotation of RDH. To characterize the RDH from *Sphingomonas* sp. PAMC 26621 grown on a minimal medium containing 1% (w/v) D-ribitol, we performed protein purification by using ammonium sulfate precipitation (30–60%) and four-step column chromatography procedures including DEAE, SP, and Mono Q columns. The proteins were first separated by using a DEAE column with buffer A, followed by the use of an SP column after desalting with buffer B and subsequent separation by using a Mono Q column with buffer C (pH 6.0). The active fractions from the Mono Q column were further separated by using a Mono Q column with buffer A (pH 8.0). The separation by the DEAE column was the most effective step in the purification process, in which the specific activity of SpRDH increased 20-fold compared to that of the ammonium sulfate precipitation step (Table 1). SpRDH appeared on an SDS-PAGE gel as a single band with a molecular weight of 39 kDa (Fig 2A, lane 7), a 25% yield, and a 123-fold purification increase (Table 1). The molecular weight of native SpRDH was estimated to be 117 kDa (trimer) by using Superdex 200 gel filtration chromatography and native-PAGE gel (Fig 2B and 2C). The trimeric structure of SpRDH with 39 kDa subunit size is distinct from that of other known RDHs that exist either as dimers or tetramers with smaller subunit sizes (25–30 kDa) [18–20, 26].

**Table 1:** Purification summary of SpRDH from *Sphingomonas* sp. PAMC 26621.

Step		Total volume (mL)	Total protein (mg)	Total activity (units)	Specific activity (units·mg^-1^)	Yield (%)	Purification (fold)
1	Crude extract	50	80	1860	23.3	100	1
2	(NH_4_)_2_SO_4_ (30–60%)	30	30	1450	48.3	78	2.1
3	DEAE column (pH 8.0)	15	1.2	1142	951.7	61.4	40.9
4	SP column (pH 5.0)	5	0.5	555	1110	30	47.7
5	Mono Q column (pH 6.0)	2	0.22	484	2200	26	94.6
6	Mono Q column (pH 8.0)	2	0.16	456	2850	24.5	122.5

### Substrate specificity, optimum pH and temperature, and thermal stability of SpRDH

**Fig 3:**
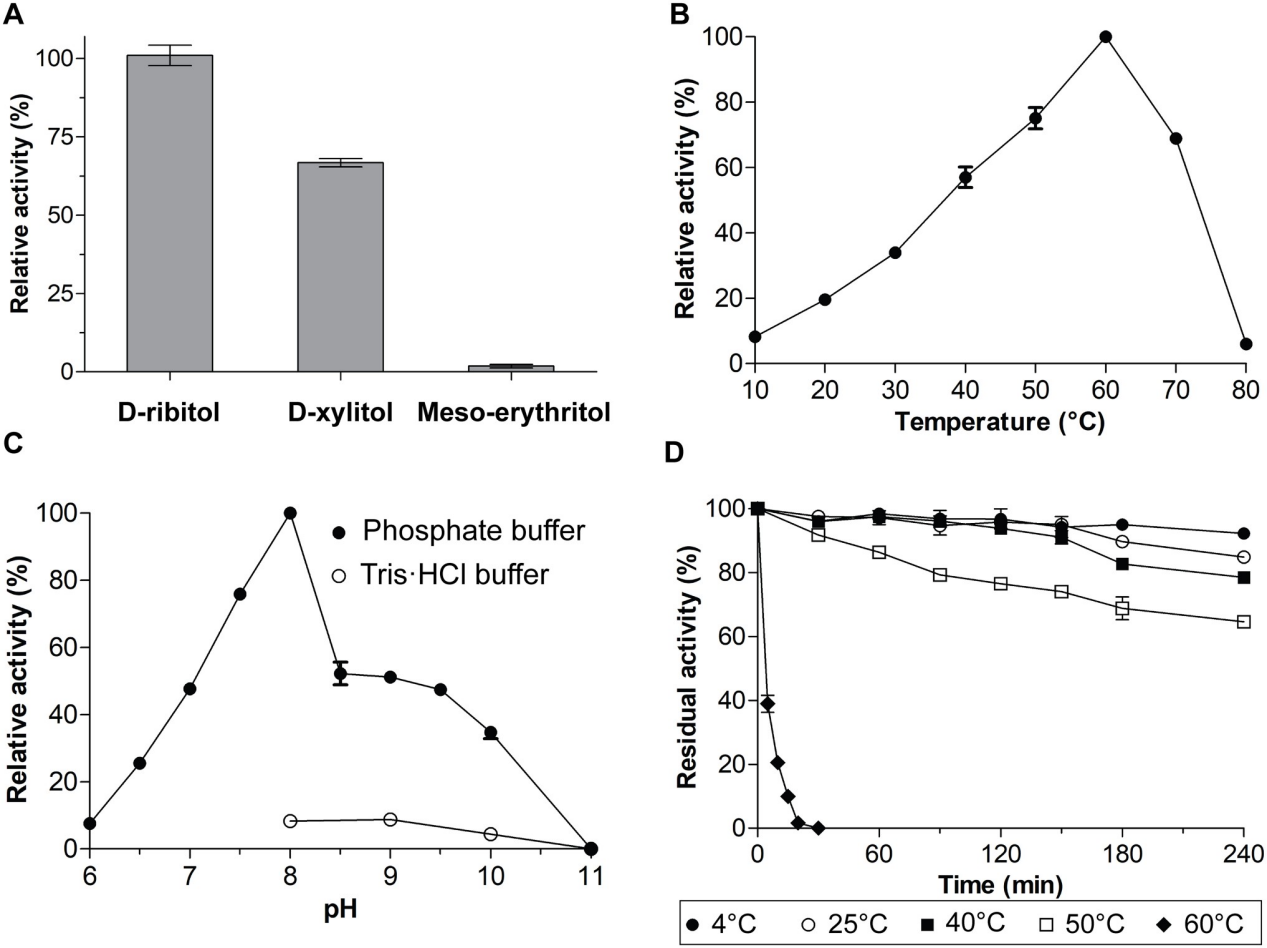
Substrate specificity, optimum temperature and pH, and thermal stability of SpRDH. (A) Substrate specificity. SpRDH activity was measured at 60°C with 40 mM of D-ribitol, D-xylitol, D-mannitol, D-sorbitol, Meso-erythritol, Myo-inositol, D-galactitol, or glycerol as substrates as described in the Materials and methods section. The SpRDH activity against D-ribitol was 100% (2800 U mg^-1^). No SpRDH activity was observed for D-mannitol, D-sorbitol, Myo-inositol, D-galactitol, and glycerol. (B) Optimum temperature. SpRDH activity was determined at various temperatures (10–80°C), as described in the Materials and methods section. (C) Optimum pH. SpRDH activity was measured at 60°C in the presence of 40 mM D-ribitol and 1 mM NAD^+^ with either 100 mM phosphate buffer (pH 6.0–11.0) or 50 mM Tris buffer (pH 8.0–11.0). (D) Thermal stability. Thermal stability of SpRDH was determined by measuring the residual activity at 60°C after incubation of the enzyme at 4–60°C for the indicated times. Enzyme activity before incubation was 100%. Each value represents the mean ± SD of three measurements.

SpRDH showed dehydrogenation activity for D-ribitol (100%), D-xylitol (66%), and Meso-erythritol (2%) with NAD^+^ as a cofactor (Fig 3A). No dehydrogenation activity was observed for D-mannitol, D-sorbitol, D-galactitol, Myo-inositol, or glycerol (Fig 3A). As SpRDH strictly used NAD^+^/NADH as a cofactor, NADP^+^/NADPH was not a cofactor. Broad substrate specificity is commonly observed for most of the known RDHs, among which D-xylitol is the most frequently preferred after D-ribitol [18, 19, 26, 27].

Many psychrophilic enzymes exhibit apparent optimum temperatures, 22–25°C higher than their physiological temperatures [28]. SpRDH exhibited an apparent optimum activity at 60°C (Fig 3B), which is 45°C higher than the optimum physiological temperature of 15°C. SpRDH showed optimum pH at 8.0 in phosphate buffer and lost its activity at pH 11.0 (Fig 3C). Notably, SpRDH showed little activity in Tris buffer (pH 8.0–11.0) (Fig 3C).

The thermal stability of SpRDH was determined by measuring residual activity at optimum operating conditions (60°C and pH 8.0) after incubation of the enzyme at various temperatures (4–60°C) and for the indicated times. SpRDH maintained dehydrogenase activity within the 4–40°C range for 2.5 h and was gradually denatured at 50°C. At 60°C, SpRDH lost its activity rapidly (Fig 3D).

### Identification of D-ribulose by HPLC

**Fig 4:**
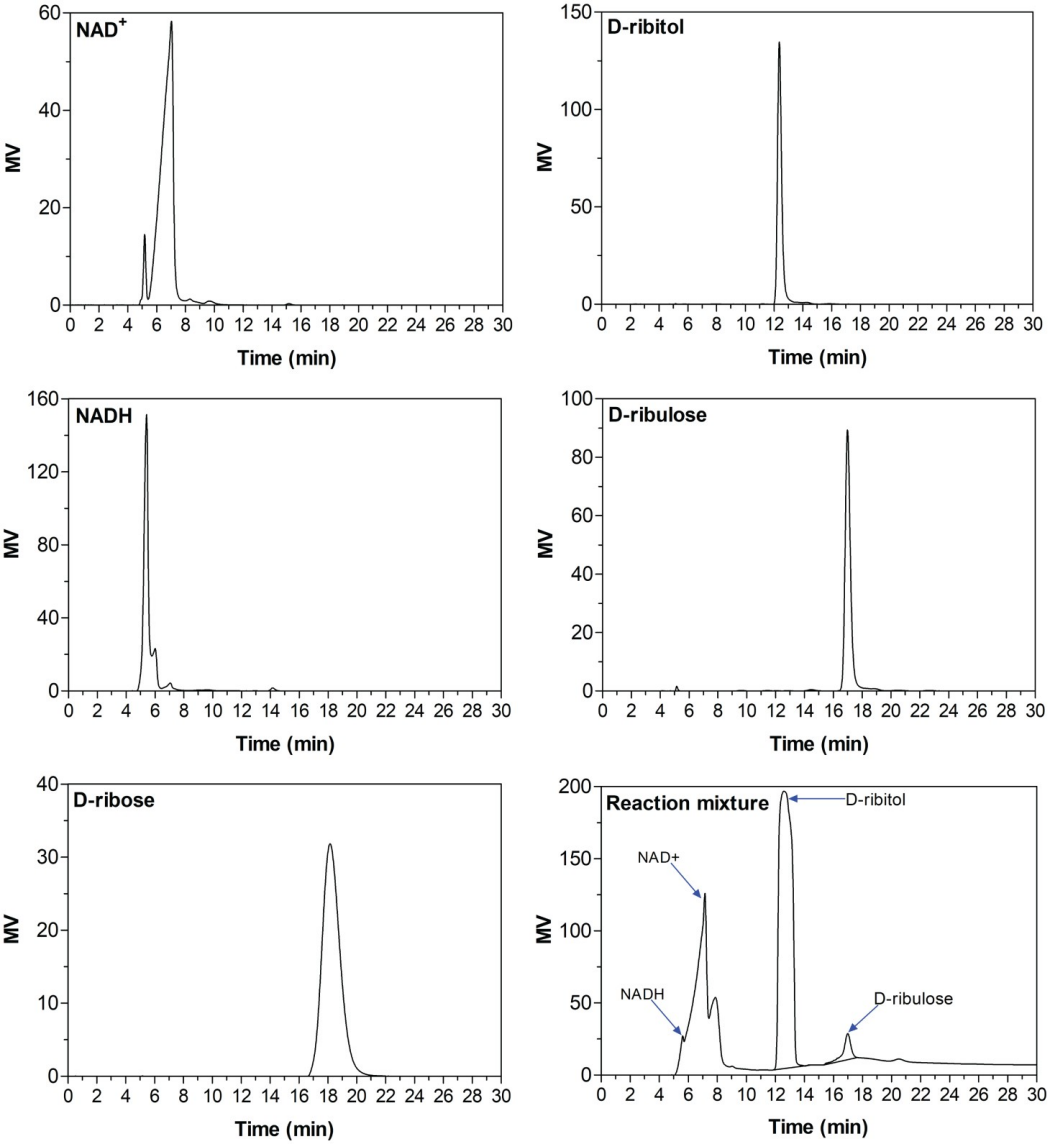
HPLC analysis of the SpRDH oxidation reaction products. The substances in the reaction mixture were analyzed using a Sugar Pak I column calibrated with authentic NAD^+^, NADH, D-ribose, D-ribulose, or D-ribitol on a Waters Alliance HPLC system.

Although previous studies showed that RDHs can use both D-ribitol and D-ribulose as substrates [19, 26, 29], no study has identified the products of RDHs. The HPLC analysis showed that SpRDH converted D-ribitol to D-ribulose in the presence of NAD^+^ (specific activity 2800 U mg^-1^ protein). The product peaks appeared in the same positions of D-ribulose and NADH in authentic graphs (Fig 4). Vice versa, SpRDH exhibited reductase activity for D-ribulose (specific activity 96 U mg^-1^ protein) in the presence of NADH. However, SpRDH could not reduce D-ribose. These results confirmed that SpRDH converts D-ribitol to D-ribulose and vice versa.

### Protein identification and amino acid sequence analysis

The amino acid sequence of SpRDH was determined by using MALDI-TOF mass spectrometry to be a putative erythritol/L-threitol dehydrogenase (NCBI ID: WP_010219437.1) (S1 Fig). However, our results presented in Figs 3 and 4 indicate that the protein is an RDH, not an erythritol/L-threitol dehydrogenase. SpRDH showed a 2% activity toward Meso-erythritol relative to that toward D-ribitol (Fig 3A). The molecular weight of SpRDH is consistent with the calculated molecular weight of the putative erythritol/L-threitol dehydrogenase (38.7 kDa) (S1 Fig).

**Fig 5:**
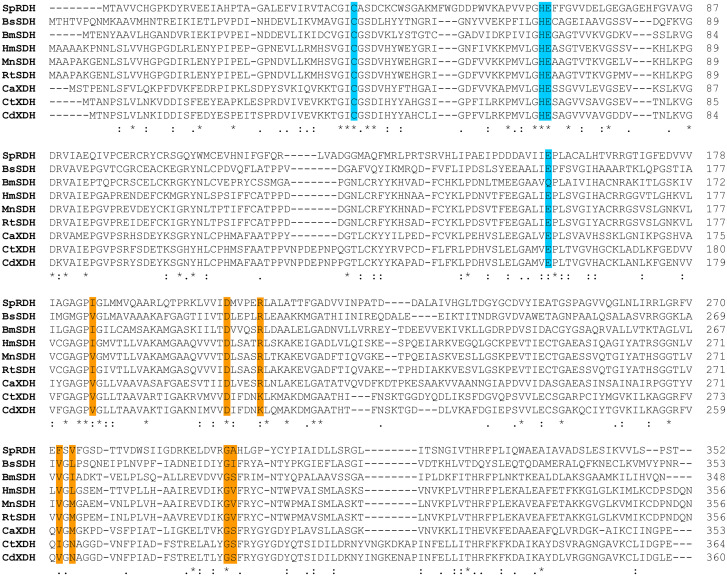
Multiple sequence alignments of SpRDH with other medium-chain SDHs and XDHs. Catalytic residues of SDH and XDH are marked in cyan, and the cofactor binding site is marked in orange. BsSDH (SDH from *Bacilus subtilis*), HmSDH (SDH from *Homo sapiens*), MnSDH (SDH from *Mus musculus*), BmSDH (SDH from *Bombyx mori*), RtSDH (SDH from *Rattus norvegicus*), CaXDH (XDH from *Candida* sp. HA 167), CtXDH (XDH from *Candida tropicali*), and CdXDH (XDH from *Candida* sp. NR20-09-22).

Multiple sequence alignment showed that SpRDH exhibited higher sequence identity with proteins from the MDR family (70–80%) (S2 Fig) and with medium-chain alcohol dehydrogenases (30–35%), including SDH and XDH from *B*. *subtilis*, *Homo sapiens*, *R*. *norvegicus*, and *C*. *tropicalis* (Fig 5). Sequence analysis indicated that SpRDH contains the zinc-binding motif ([GHE]xx[G]xxxxx[G]xx[A]) as has been observed in other zinc-dependent alcohol dehydrogenases (S2 Fig) [30]. The catalytic residues of medium-chain SDH and XDH were also conserved in the amino acid sequence of SpRDH (Cys37, His65, Glu66, and Glu157) (Fig 5). In contrast, all known short-chain RDHs have catalytic residues consisting of Ser, Tyr, and Lys (S3 Fig). SpRDH had low sequence identity (15–17%) with RDHs from *Z*. *mobilis*, *K*. *aerogenes*, and *P*. *alcalifaciens*, all of which belong to the SDR family [18–20, 29, 31] (S3 Fig).

### Metal ion analysis

All short-chain RDHs and medium-chain SDHs and XDHs have been shown to be metal ion-dependent enzymes [18–20, 26, 30, 32]. To investigate the effects of metal ions on the activity of SpRDH, EDTA was used at 1 mM and 10 mM in the SpRDH reaction mixture. The results showed that EDTA did not affect SpRDH activity (Table 2). Consistent with that result, the ICP-MS analysis of metal ions on SpRDH showed no appreciable amounts of divalent metal ions (Zn^2+^, Mg^2+^, Ni^2+^, Co^2+^, Mn^2+^, Cu^2+^, and Fe^2+^). These results suggest that divalent metal ions are not required for SpRDH activity.

**Table 2:** Metal ion analysis.

A. Effect of EDTA on SpRDH activity		
	Relative activity (%)^a^	
	1 mM	10 mM
None	100 ± 1	100 ± 1
EDTA	100 ± 0.8	99 ± 0.5
B. ICP-MS analysis		
Metal ion	Concentration (μg L^-1^)	Metal content (mol/mol SpRDH subunit)
Mg^2+^	3.0	0.012
Mn^2+^	< 1.0	< 0.002
Fe^2+^	< 1.0	< 0.002
Co^2+^	< 1.0	< 0.002
Ni^2+^	< 1.0	< 0.002
Cu^2+^	< 1.0	< 0.0015
Zn^2+^	< 1.0	< 0.0015

^a^Each value represents the mean ± SD of three independent experiments.

### Kinetic parameters

Initial velocities were determined in a standard reaction mixture containing 10–160 mM D-ribitol, while the NAD^+^ concentration was held constant at 0.05, 0.1, 0.2, 0.5, or 1 mM in 100 mM potassium phosphate (pH 8.0). SpRDH had *K*_m_ values of 8.5 mM and 0.3 mM for D-ribitol and NAD^+^, respectively (Table 3). The catalytic rate (*k*_cat_) value of the SpRDH oxidation reaction was 29 s^−1^ (Table 3). The *K*_m_ value of SpRDH for D-ribitol was similar to those of other RDHs [18–20, 26]. However, the *K*_m_ value of SpRDH for NAD^+^ was higher than those of most other RDHs (*i*.*e*., 0.04–0.16 mM) (Table 3). Taken together, the results indicate that SpRDH converts D-ribitol into D-ribulose with a relatively high catalytic efficiency (*k*_cat_/*K*_m_ = 3.5 mM^−1^ s^−1^) (Table 3).

**Table 3:** Biochemical properties of SpRDH and other RDHs.

Organism	M.W. (kDa)	Quaternary structure	Optimum pH	Kinetics					Reference
				*K*_m_, D-ribitol (mM)	*K*_m_, NAD^+^ (mM)	*k*_cat_, (s^-1^)	*k*_cat_/*K*_m_, D-ribitol (mM^−1^ ·s^−1^)		
*Sphingomonas* sp. PAMC 26621	39	Trimer	8.0	8.5±1.2	0.3±0.02	29±0.7	3.5	Purified	This study
*Enterobacter agglomerans*	25	Tetramer	9.0	32.2	2.36	NR	NR	Purified	[27]
*Klebsiella aerogenes*	27	Tetramer	11.0	5	0.1	NR	NR	Purified	[31]
*Rhodobacter sphaeroides*	25	Dimer	9.0	6.3	0.08	NR	NR	Purified	[26]
*Gluconobacter suboxydans*	25	Tetramer	9.5–10.5	1.2	0.08	NR	NR	Purified	[33]
*Zymomonas mobilis*	28	Dimer	9.5	11.8	0.18	4.83	0.4	Recombinant	[19]
*Providencia alcalifaciens*	27	Dimer	10.0	13.9	0.04	10	0.7	Recombinant	[18]
*Enterobacter aerogenes*	25	Tetramer	11.0	10.3	0.16	318	30.9	Recombinant	[20]

MW: molecular weight, NR: Not reported

## Discussion

In the present study, based on the observation that the bacterium *Sphingomonas* sp. PAMC 26621 isolated from the Arctic lichen *Cetraria* sp. utilized D-ribitol as its sole carbon source among the polyols examined (Fig 1), we proceeded to purify and characterize a novel RDH (SpRDH) from the bacterium. The lichen *Cetraria* sp. is commonly associated with several green algae of the *Trebouxia* genus as a photobiont [34], releasing ribitol as a mobile carbohydrate within the lichen thalli [5, 12]. Among the bacteria associated with lichens, *Alphaproteobacteria*, including the genus *Sphingomonas*, are frequently detected [1, 2, 14], especially in the Arctic and Antarctic lichens [35]. The *sphingomonads* do not have phosphofructokinase (*pfk*), which is required for the Embden-Meyerhof-Parnas pathway; instead, they use the Entner-Doudoroff pathway for glucose metabolism [36]. Many sugar alcohols, such as D-ribitol, D-arabitol, and D-xylitol, enter the pentose phosphate pathway for carbon metabolism. The SpRDH operon of *Sphingomonas* sp. PAMC 26621 genome contains a putative ABC transporter and a ribulokinase (S4 Fig). The ribulokinase converts D-ribulose to D-ribulose 5-phosphate for entry into the pentose phosphate pathway.

RDH is frequently detected in many *Alphaproteobacteria* [18, 19, 26, 33], indicating their ability to metabolize ribitol as an energy source [14]. However, SpRDH is different from other known RDHs, all of them belonging to the SDR family, in several aspects. First, SpRDH shares high sequence similarity to the polyol dehydrogenases of the MDR family, including medium-chain SDHs and XDHs. Moreover, the molecular weight of SpRDH (39 kDa) is larger than that of short-chain RDHs (25–30 kDa), and the trimeric structure of native SpRDH is different from the dimeric or tetrameric structures of short-chain RDHs. Second, multiple sequence alignments of SpRDH with other MDR family enzymes revealed that the catalytic amino acid residues of SpRDH (Cys37, His65, and Glu66 and Glu157) differ from those of short-chain RDHs (Ser, Tyr, and Lys). The basic pK_a_ values of the R group of the Tyr and Lys residues of short-chain RDHs shift their optimum pH toward an alkaline condition (pH 9.0–11.0). The optimum pH of SpRDH in phosphate buffer (pH 8.0) is reflective of the different catalytic residues of SpRDH. Of further interest, SpRDH did not exhibit activity in Tris buffer, whereas short-chain RDHs from *Z*. *mobilis* and *E*. *aerogenes* are active in Tris buffer (pH 8.5–10.5) [19, 20]. Moreover, the cofactor binding residues of SpRDH were also different from those of other MDR family members. Third, while the catalytic activities of short-chain RDHs are metal ion-dependent, particularly Zn^2+^, SpRDH did not require divalent metal ions as was demonstrated by ICP-MS analysis and by EDTA treatment on SpRDH. Despite the Zn^2+^-independence, SpRDH exhibits a zinc-binding motif ([GHE]xx[G]xxxxx[G]xx[A]) (Fig 5). Thus, further studies on the catalytic mechanisms of SpRDH should be pursued.

External ribitol was reported to inhibit the transport of ribitol from the algae to the fungi in lichen thalli [5, 37], leading to a decrease in the potential quantum yield of photosystem II [38]. From the viewpoint of lichen symbiosis, the *sphingomonads* can obtain benefits from that symbiosis as they utilize external ribitol not only for carbon sources but also to accelerate the transport of ribitol within the lichen [14]. On the other hand, while the photosynthetic process of lichen algae can be inhibited by low temperatures and osmotic stress [39], internal ribitol in the lichen can serve as a cryoprotectant and osmoprotectant by blocking the formation of ice crystals through steric mismatching of the hydrogen bonds formed between ribitol and water [11, 38], thus stabilizing the structure of photosystem II and leading to increased photosynthesis in lichens [25]. The production of ribitol by algae can be stimulated in the symbiotic state [5]; thus, the green algae and mycobionts also benefit from the symbiosis with *sphingomonads*.

In conclusion, we demonstrated that SpRDH is the first RDH that belongs to the MDR family. Further studies on the structure/function relationships and the catalytic mechanism of SpRDH will help elucidate the roles of polyol dehydrogenases in lichen symbiosis and illuminate their evolutionary differences from other non-lichen polyol dehydrogenases.

## Supporting information

S1 Fig Proteomic analysis. (PDF)

S2 Fig Multiple sequence alignments of SpRDH with other proteins belonging to the MDR family. (PDF)

S3 Fig Multiple sequence alignments of SpRDH with short-chain RDHs. (PDF)

S4 Fig The structure of SpRDH operon. (PDF)

S1 Raw images (PDF)
