# Supplementary material for: Purification and characterization of a novel medium-chain ribitol dehydrogenase from a lichen-associated bacterium Sphingomonas sp
Source: PLoS One. 2020 Jul 8;15(7):e0235718. doi: 10.1371/journal.pone.0235718 (PMC7343156; doi:10.1371/journal.pone.0235718)
Supplement: S1 Fig — (PDF) [file pone.0235718.s001.pdf]

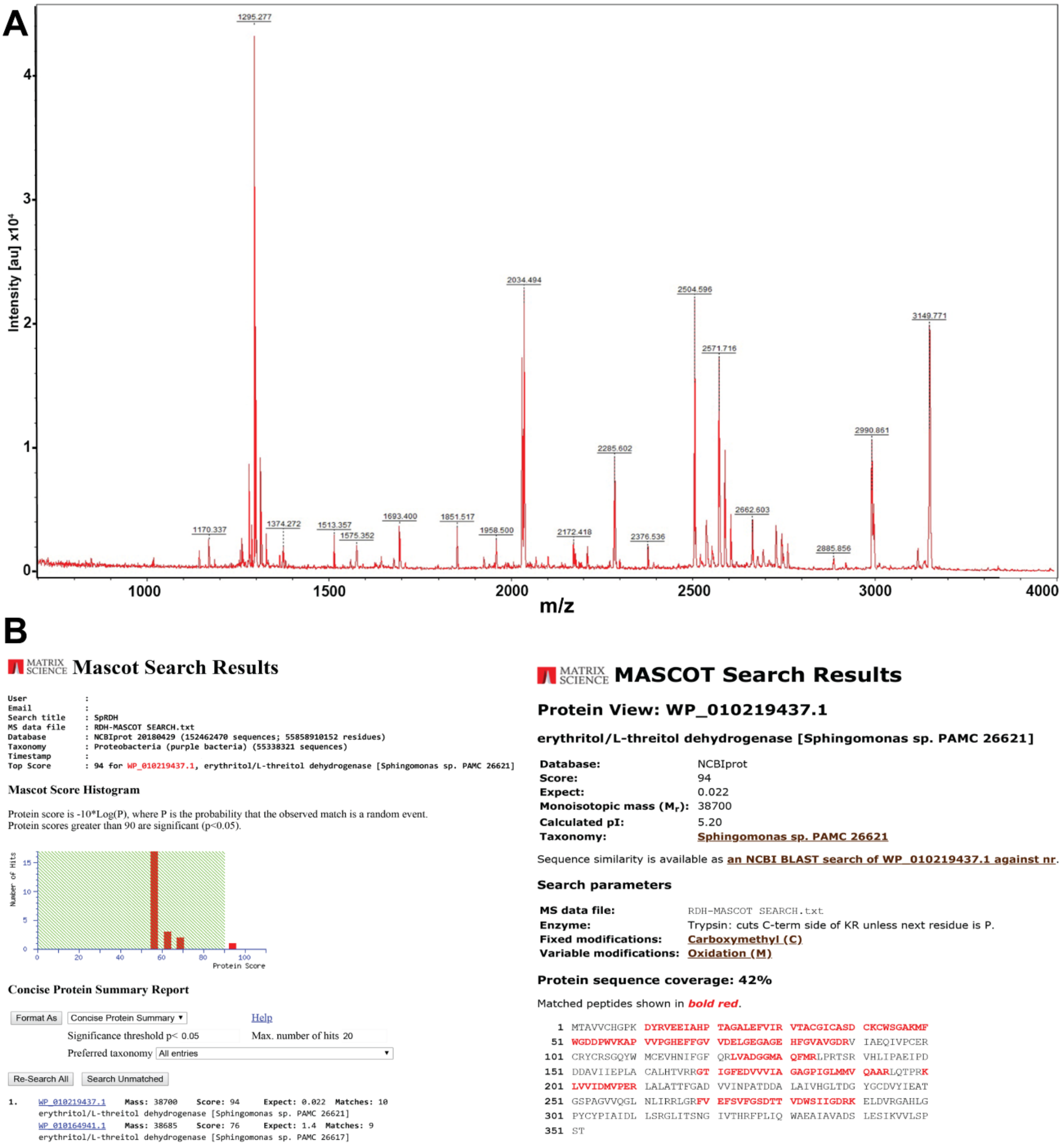

S1 Fig Proteomic analysis. (A) MALDI-TOF analysis results of the trypsin-digested proteins samples of SpRDH. (B) Mascot analysis of the MS/MS peaks of the SpRDH sample.
