# Supplementary material for: Purification and characterization of a novel medium-chain ribitol dehydrogenase from a lichen-associated bacterium Sphingomonas sp
Source: PLoS One. 2020 Jul 8;15(7):e0235718. doi: 10.1371/journal.pone.0235718 (PMC7343156; doi:10.1371/journal.pone.0235718)
Supplement: S2 Fig — (PDF) [file pone.0235718.s002.pdf]

```
SpRDH   MTAVVCHGPKDYRVEEIAHPTAGALEFVIRVTACGICASDCKCWSGAKMFWG-DDPWVKAPVVPGHEFFGVVDELGEGAGEHFGVAVG   87
BsSDH   MTAVVCHAPEDYRVERVTKPRARARELVIRIGACGICASDCKCHAGAKMFWGGPSPWVKAPVIPGHEFFGYVDELGEGAAEHFGVALG   88
CaADH   MRAIVCHGPEDYRVEDVSRPRPGDREMIIRIGACGVCASDCKCWSGAKMFWGGENPWVKAPVVPGHEFFGYVDELGAGAAEHFGVAMG   88
CpADH   MTAIVCRAPKDYRVERVQRPSAARNELVIRIAACGICASDCKCWSGAKMFWGGPNPWVKAPVIPGHEFFGYVEELGEGAQEHFGVQKG   88
GrADH   MTAIVCHAPKDYRVEQVARPVPGPREIVIRIGACGICASDCKCWSGAPMFWEGKQPYVKPPVIPGHEFFGYVDELGEGADAHFGVGVG   88
MvIDH   MTAIVCHGPEDYRVQQVDRPVPEEREMVLRIAACGICASDCKCWSGAKMFWGGDNPYVKPPVIPGHEFYGVVDELGPGAAEHFGVGIG   88
PaADH   MTAIVCHAPKDYRVEQVSKPRAGAHELVIRIAACGICASDCKCHSGAKMFWGGPSPWVKAPVIPGHEFFGFVEEIGEGAADHFGVKMG   88
PbADH   MTAIVCHAPKDYRVEQVSKPTARAHELVIRIAACGICASDCKCHSGAKMFWGGPSPWVKAPVIPGHEFFGFVEQIGEGAADHFGVKLG   88
PcADH   MTAIVCRAPKDYRVEQVARPRAGRNELVIRIAACGICASDCKCWSGAKMFWGGPNPWVKAPVIPGHEFFGYVEELGEGAAEHFNVQLG   88
TcADH   MTAVVCHAPKDYRVERVSRPKAGRNELVIRIGACGVCASDCKCWSGARMFWGGPNPWVKAPVIPGHEFFGYVEELGEGAAEHFGVRVG   88
        * *:**:.*:****: : :*     *:::*: ***:******* :** ***   .*:** **:*****:* *:::* **  **.*  *

SpRDH   DRVIAEQIVPCERCRYCRSGQYWMCEVHNIFGFQRLVADGGMAQFMRLPRTSRVHLIPAEIPDDDAVIIEPLACALHTVRRGTIGFED   175
BsSDH   ERVIAEQIVPCGQCRYCKHGQYWMCEVHNIFGFQREVADGGMAEYMRIPPTAIVHKIPAGISVEDAAIIEPLACAIHTVNRGDIQFDD   176
CaADH   DRVIAEQIVPCERCRYCRSGHYWMCEVHNIFGFQREVAEGGMAEFMRIPATARVHRIPDGISLDDAAIIEPLACAIHTVNRADVQLDD   176
CpADH   DRVIAEQIVPCAKCRYCKSGEYWMCEVHNIFGFQREVADGGMAEYMRFPPTAIVHKIPDGISLEDAAIIEPLACAIHTVNRGDIQLSD   176
GrADH   DMVIAEQIVPCDKCRYCRSGQYWMCEVHNIYGFQREVADGGMAEYMRIGETARLHKIPAEMPADDAGIIEPLACAIHTVQRGDVQLDD   176
MvIDH   ERVIAEQIVPCDQCRFCRSGQYWMCEVHNIFGFQRVVADGGMAEYMRIPRTARVHKIDEGISANDAAIIEPLACAIHTVNRGDIQLDD   176
PaADH   DRVIAEQIVPCGKCRYCKSGQYWMCEVHNIFGFQREVADGGMAEYMRIPPTAIVHKIPDGISLEDAAIIEPLACAIHTVNRGEVQLDD   176
PbADH   DRVIAEQIVPCGKCRYCKSGQYWMCEVHNIFGFQREVADGGMAEYMRIPPTAIVHKIPDGISLEDAAIIEPLACAIHTVNRGEVQLDD   176
PcADH   ERVIAEQIVPCDKCRYCRSGQYWMCEVHNIFGFQREVADGGMAQYMRFPPTAIVHKIPLGVSLEDAAIIEPLACAIHTVNRGDIQLDD   176
TcADH   ERVIAEQIVPCGKCRYCKSGQYWMCEVHNIFGFQREVADGGMAEYMRIPPTAIVHKIPDGISIEDAAIIEPLSCAIHTVNRGDIQLDD   176
        : ********* :**:*: *.*********:**** **:****::**:  *: :* *   :  :** *****:**:***.*. : :.*

SpRDH   VVVIAGAGPIGLMMVQAARLQTPRKLVVIDMVPERLALATTFGADVVINPATDDALAIVHGLTDGYGCDVYIEATGSPAGVVQGLNLI   263
BsSDH   VLVIAGAGPLGLMMTQVAKLRTPKRLIVIDLVDERLALARDYGADVTINPARDDALSIVHALTGGYGCDVYIETTGVPAGVTQGLALI   264
CaADH   VVVIAGAGPIGLMMVQVARLKTPKKLVVIDMVPERLELARRFGADVVINPREEDAWPIVSGLTGGYGCDAYIEATGSPSGVEQGLTLI   264
CpADH   VVVIAGAGPLGLMMTQVAHLKTPKKLVVIDLVEERLALAREYGADITINPKTDDALETIRSITDRYGCDVYIETTGAPAGVTQGLELI   264
GrADH   VVVIAGAGPIGLMMVQVAKLKTPRKLVVIDMVAERRELALKFGADVAIDPANEDAAAIVKGLTGGYGCDVYIEATGAPAGVTQGLELI   264
MvIDH   VVVIAGAGPIGLMMVQVTKLKTPKLLIAIDMVDERLQLAKTFGADVVINPREQDALTVVRSLTDGYGCDVYIEATGSPSGVEQGLNLI   264
PaADH   VVVIAGAGPLGLMMTQIAHLKTPKKLVVIDLVEERLALAREYGADVTINPKQDDALAIIHSLTDGYGCDVYIETTGAPIGVNQGMDLI   264
PbADH   VVVIAGAGPLGLMMTQIAHLKTPKKLVVIDLVEERLALAREYGADVTINPKQDDALAIVHSLTDGYGCDVYIETTGAPIGVNQGMDLI   264
PcADH   VVVIAGAGPLGLMMVQVAHLKTPKKLVVIDLVDERLALAREYGADVTINPASDDALGVVRSLTDQYGCDVYIETTGSPSGVTQGLDLI   264
TcADH   VVVIAGAGPLGLMMTQVAHLKTPKKLVVIDLVDERLALAREYGADVTINPKNDDALAIVRSLTDQYGCDVYIETTGAPGGVTQGLELI   264
        *:*******:****.* ::*:**: *:.**:* **  **  :***:.*:*  :**   : .:*. ****.***:** * ** **: **

SpRDH   RRLGRFVEFSVFGSDTTVDWSIIGDRKELDVRGAHLGPYCYPIAIDLLSRGLITSNGIVTHRFPLIQWAEAIAVADSLESIKVVLSPST  352
BsSDH   RKLGRFVEFSVFGKDTTADWSIIGDRKELDVRGAHLGPYCYPVAIDLLARGLVTSNGIVTHGFTLDEWDEAIRVANSLDSIKVLMRP--  351
CaADH   RKLGRFVEFSVFGAPATVDWSIIGDRKELDVRGAHLGPYCYPIAIDLLARGLVTSQGIVTHRYGLEAWDEAIRVANSLDSIKVLMQPEN  353
CpADH   RKLGRFVEFSVFGADTTVDWSIIGDRKELDVRGAHLGPYCYPIAIDLLARGLVTSKGIVTHGFTLEEWDEAIAVANSLDSIKVLLKPK-  352
GrADH   RKLGRFVEFSVFGAPTTADWSIIGDRKELDIRGAHLGPYCYPIAIDLLMRGLVTSKGIVTHEYPLEAWDEAIGMAYGLDSIKVLLKP--  351
MvIDH   RKLGRFVEFSVFGAPTTVDWSIIGDRKELDIRGAHLAPYCYPIAINLLSRGLVTSKGIVTHRYRLEEWDTAIAVANSLESIKVLMDATS  353
PaADH   RKLGRFVEFSVFGADTTLDWSVIGDRKELDVRGAHLGPYCYPIAIDLLARGLVTSKGIVTHGFSLEEWDEAIKIANSLDSIKVLLKP--  351
PbADH   RKLGRFVEFSVFGADTTLDWSVIGDRKELDVRGAHLGPYCYPIAIDLLARGLVTSKGIVTHGFSLEEWDEAIKVANSLDSIKVLLKP--  351
PcADH   RKLGRFVEFSVFGSDTTTDWSIIGDRKELDVRGAHLGPYCYPIAIDLLARGLVTSKGIVTHGFSLEQWDEAIKVANSLDSIKVLLKPKT  351
TcADH   RKLGRFVEFSVFGAETTVDWSIIGDRKELDVRGAHLGPYCYPIAIDLLARGLVTSKGIVTHGFSLEEWDEAIKVANSLDSIKVLMRPK-  352
        *:***********  :* ***:********:*****.*****:**:** ***:**:***** : *  *  ** :* .*:****::
```

S2 Fig Multiple sequence alignments of SpRDH with other proteins belonging to MDR superfamily. BsSDH (sorbitol dehydrogenase from *Burkholderia singularis*), CaADH (alcohol dehydrogenase [ADH] from *Chelatococcus asaccharovorans*), CpADH (ADH from *Caballeronia pedi*), GrADH (ADH from *Geminicoccus roseus*), MvIDH (iditol 2-dehydrogenase from *Microvirga vignae*), PaADH (ADH from *Paraburkholderia aromaticivorans*), PbADH (ADH from *Paraburkholderia bryophila*), PcADH (ADH from *Paraburkholderia caribensis*), TcADH (ADH from *Trinickia caryophylli*). Zinc-binding motif, [GHE]xx[G]xxxxx[G]xx[A], is indicated in a red box.
