# Supplementary material for: Purification and characterization of a novel medium-chain ribitol dehydrogenase from a lichen-associated bacterium Sphingomonas sp
Source: PLoS One. 2020 Jul 8;15(7):e0235718. doi: 10.1371/journal.pone.0235718 (PMC7343156; doi:10.1371/journal.pone.0235718)
Supplement: S3 Fig — (PDF) [file pone.0235718.s003.pdf]

```
SpRDH  MTAVVCHGPKDYRVEEIAHPTAGALEFVIRVTACGICASDCKCWSGAKMFWGDDPWVKAPVVPGHEFFGVVDELGEGAGEHFGVAVGDRVIAEQIV  96
ZmRDH  ------------------------------------------------------------------------------------------------  0
RsRDH  ------------------------------------------------------------------------------------------------  0
PaRDH  ------------------------------------------------------------------------------------------------  0
KaRDH  ------------------------------------------------------------------------------------------------  0
EaRDH  ------------------------------------------------------------------------------------------------  0

SpRDH  PCERCRYCRSGQYWMCEVHNIFGFQRLVADGGMAQFMRLPRTSRVHLIPAEIPDDDAVIIEPLACALHTVRRGTIGFEDVVV--IAGAGPIGLMMV  190
ZmRDH  -------------------------------------MIPR----------------------PDHGEKSYRGSGKLQGRKALITGGDSGIGRATA  37
RsRDH  ------------------------------------------------------------------------MAELMQGKVAAITGAASGIGLECA  24
PaRDH  ------------------------------------------------------------------------MAISLENKVAAITGAASGIGLECA  24
KaRDH  -----------------------------------------------------------------MNHSVSSMNTSLSGKVAAITGAASGIGLECA  31
EaRDH  ------------------------------------------------------------------------MNTSLSGKVAAVTGAASGIGLECA  24
                                                                                   :..  .   .. . **   .

SpRDH  QAARLQTPRKLVVIDMVPERLALATTFGADVVINPATDDALAIVHGLTDGYGCDVYIEATGSPAGVVQGLNLIRR-LGRFVEFSVFGSDTTVDWSI  285
ZmRDH  IA-YAREGADIVINYLPQEEPD-AR--EVVALLQGEGHKVFALPGDLRNENFCVQLVQEASKRLGGL---DILANIAGHQHYNESILTLSTADFDD  126
RsRDH  RT-LVAEGATVVLIDRAED--------RLKALCAEIGPRALPLVVDLLDGPQVSGMLPRILELAGSL---DIFHANAGAYIG-GQVAEGDPDAWDR  107
PaRDH  RT-LLKAGAKVVLIDRAEE--------RLNQLVAELGDSAIPLVVDLMKPEQVDGMLDAILAKAGRL---DIFHANAGAYIG-GPVAEGDPDVWDK  107
KaRDH  RT-LLGAGAKVVLIDREGE--------KLNKLVAELGENAFALQVDLMQADQVDNLLQGILQLTGRL---DIFHANAGAYIG-GPVAEGDPDVWDR  114
EaRDH  KT-MLGAGAKVVLIDREGE--------KLNKIVAELGENAFALQVDLMQGDQVDKIIDGILQLAGRL---DIFHANAGAYIG-GPVAEGDPDVWDR  107
        :        :*:     :            :       .: :  .* .       :       * :   :::    *       .       :.

SpRDH  IGDRKELDVRGAH-LGPYCYPIAIDLLSRGLIT---SNGIVTHRFPLIQWAEAIAVADSLESIKVVLSPST-------------------------  352
ZmRDH  TLK---TNDYAMFWLCKEALKI--MPAGSAIVNTSSKQGYSTPAILLDYATSKAAIANFTRALAIQLAPRGIRVNAVAPGPFWTPLQVSGGQPASA  217
RsRDH  MLN---LNINAAFRSVHAVLPYMIERKSGDILFTSSVAGVVPVVWEPIYTASKFAVQAFVHSTRRQVAPHGVRVGAVLPGPVVTALLDDW--PKAK  198
PaRDH  VLN---LNINAAFRSVRAVLPHFIEQKSGDVLFTSSIAGMVPVIWEPIYTASKFAVQAFVHSTRRQVSQYSVRVGAVLPGPVVTALLDDW--PKEK  198
KaRDH  VLH---LNINAAFRCVRSVLPHLIAQKSGDIIFTSSIAGVVPVIWEPVYTASKFAVQAFVHTTRRQVAQYGVRVGAVLPGPVVTALLDDW--PKAK  205
EaRDH  VLH---LNTNAAFRCVRSVLPHMIAQKSGDIIFTSSIAGVVPVIWEPIYTASKFAVQAFVHTTRRQVSQHGVRVGAVLPGPVVTALLDDW--PKEK  198
         .    :  . .              .  ::      *           :.  *:    .:    ::

SpRDH  ------------------------------------------------  352
ZmRDH  YSQFGANTPVKRPGQPVEIAPVYVFLACAENSYVTGEVYGETGCAQIF  265
RsRDH  MEEALANG---SLMQPKEVAEAVLFMLSRPKGVVIRDLVILPHSVDI-  242
PaRDH  MEEALANG---SLMQPIEVAEAVLFMLTRPKNVTIRDLVILPNSVDL-  242
KaRDH  MDEALANG---SLMQPIEVAESVLFMVTRSKNVTVRDIVILPNSVDL-  249
EaRDH  MEEALANG---SLMQPIEVAESVLFMVTRSKNVTVRDLVILPNSVDL-  242
```

S3 Fig Multiple sequence alignments of SpRDH with short-chain RDHs. The catalytic amino acid residues of SpRDH (Cys37, His65, Glu66, and Glu157) differ from those of short-chain RDHs (Ser, Tyr, and Lys) colored in cyan. *Z. mobilis* (ZmRDH), *R. sphaeroides* (RsRDH), *P. alcalifaciens* (PaRDH), *K. aerogenes* (KaRDH), and *E. aerogenes* (EaRDH).
