# Supplementary material for: Purification and characterization of a novel medium-chain ribitol dehydrogenase from a lichen-associated bacterium Sphingomonas sp
Source: PLoS One. 2020 Jul 8;15(7):e0235718. doi: 10.1371/journal.pone.0235718 (PMC7343156; doi:10.1371/journal.pone.0235718)
Supplement: S4 Fig — (PDF) [file pone.0235718.s004.pdf]

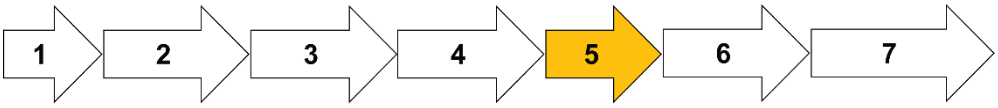

S4 Fig The structure of SpRDH operon. 1. Transcriptional regulator (WP_010219443.1), 2. ABC transporter substrate-binding protein (WP_010219442.1), 3. ABC transporter ATP-binding protein (WP_010219441.1), 4. Ribose ABC transporter permease (WP_081504296.1), 5. SpRDH (WP_010219437.1), 6. Ribulokinase (WP_010219436.1), 7. Carbohydrate porin (WP_010219434.1).
