# Supplementary material for: Purification and characterization of a novel medium-chain ribitol dehydrogenase from a lichen-associated bacterium Sphingomonas sp
Source: PLoS One. 2020 Jul 8;15(7):e0235718. doi: 10.1371/journal.pone.0235718 (PMC7343156; doi:10.1371/journal.pone.0235718)

The figures were captured by scanning on an Epson Perfection 4490 scanner

Figure 2A: Silver-stained SDS gel after protein purification

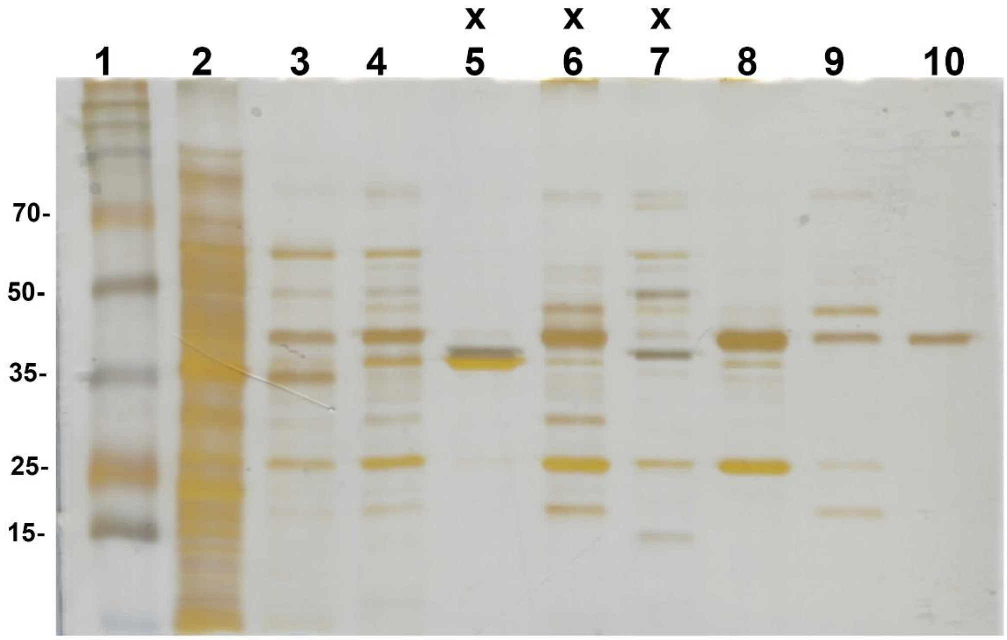

Figure 2C: Native-PAGE analysis of SpRDH

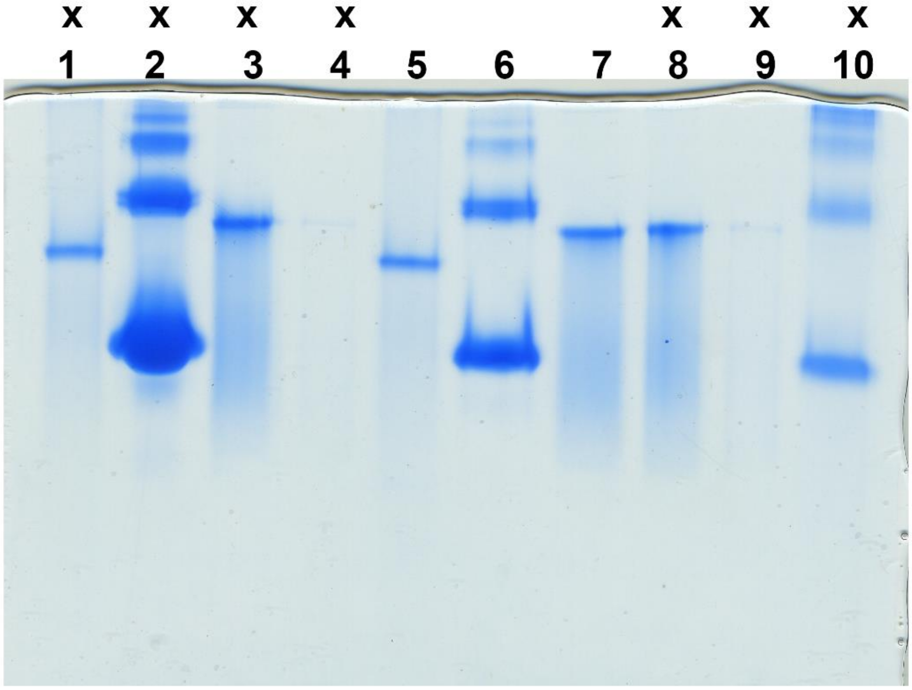

Supplement: S1 Raw images — (PDF) [file pone.0235718.s005.pdf]
